# Abnormal static and dynamic functional connectivity of resting-state fMRI in multiple system atrophy

**DOI:** 10.18632/aging.103676

**Published:** 2020-08-27

**Authors:** Weimin Zheng, Yunxiang Ge, Shan Ren, Weizheng Ran, Xinning Zhang, Wenyang Tian, Zhigang Chen, Weibei Dou, Zhiqun Wang

**Affiliations:** 1 Department of Radiology, Aerospace Center Hospital, Beijing 100049, China; 2 Department of Electronic Engineering, Beijing National Research Center for Information Science and Technology (BNRist), Tsinghua University, Beijing 100084, China; 3 Department of Neurology, Dongfang Hospital, Beijing University of Chinese Medicine, Beijing 100078, China; 4 Beijing University of Chinese Medicine, Beijing 100029, China

**Keywords:** multiple system atrophy, dynamic functional connectivity, graph theory attribute, receiver operating characteristic, ventral anterior cingulate cortex

## Abstract

In order to explore the topological alterations in functional brain networks between multiple system atrophy (MSA) patients and healthy controls (HC), a new joint analysis method of static and dynamic functional connectivity (FC) is proposed in this paper. Twenty-four MSA patients and twenty HCs were enrolled in this study. We constructed static and dynamic brain networks from resting-state fMRI data and calculated four graph theory attributes. Statistical comparisons and correlation analysis were carried out for static and dynamic FC separately before combining both cases. We found decreased local efficiency (LE) and weighted degree (WD) in cerebellum from both static and dynamic graph attributes. For static FC alone, we identified increased betweenness centrality (BC) at left dorsolateral prefrontal cortex, left Cerebellum_Crus9 and decreased WD at Vermis_6. For dynamic FC alone, decreased BC, clustering coefficients and LE at several cortical regions and cerebellum were identified. All the features had significant correlation with total UMSARS scores. Receiver operating characteristic analysis showed that dynamic features had the highest area under the curve value. Our work not only added new evidence for the underlying neurobiology and disrupted dynamic disconnection syndrome of MSA, but also proved the possibility of disease diagnosis and progression tracking using rs-fMRI.

## INTRODUCTION

Multiple system atrophy (MSA) is a progressive neurodegenerative disorder manifested by parkinsonism, cerebellar ataxia syndrome, as well as autonomic nervous dysfunction [1]. Pathologically, it is characterized by alpha synuclein-positive glial cytoplasmic inclusions (GCIs), which lead to the degeneration and death of neurons, finally result in the brain atrophy in several specific regions including the striatum, cerebellum, and olivopontine structures [2–4]. However, the mechanism of MSA remains unclear at present. The main hypothesis of MSA pathophysiology suggested that the disruption of functional connectivity (FC) among specific brain regions, caused by alpha synuclein-positive GCI, might contribute to the clinical performances of MSA.

Recent advances in neuroimaging techniques provide the opportunity to study the disconnections (i.e., disruption of functional connectivity) in MSA in vivo. The resting-state functional magnetic resonance imaging (rs-fMRI) can explore statistical correlations of spontaneous activities among functional correlated brain regions and different brain networks [5, 6]. In the previous studies, researchers found the disruption of the striatal-thalamo-cortical (STC) network, default mode network (DMN), visual associated network [7], cerebello-thalamo-cortical (CTC) network [8], as well as motor network [9] in MSA patients, which were associated with the movement dysfunction. Furthermore, recent MSA studies revealed the altered functional connectivity of DMN, sensorimotor network, visual associated cortices and cerebellum [10, 11], which showed close correlation with structure atrophy and perfusion dysfunction. These studies provided evidence for the hypothesis of “disconnection syndrome”, suggesting that the accumulation of alpha-synuclein GCIs of the MSA may destroy the specific networks and finally result in associated clinical dysfunction [12]. Besides resting state networks, graph theoretical analysis is another method to study brain networks. Researchers found altered network topology and graph theory attributes by diffusion tensor tractography [13] and rs-fMRI [14, 15]. Despite these advances, previous studies mostly assumed that the functional connectivity was constant during the MRI scanning, ignoring its dynamic nature [16–18].

Compared to static FC, dynamic FC offers the chance to investigate the rs-fMRI time series on a much finer scale (e.g., at specific time points or within predefined time windows), which brings two exclusive advantages. On the one hand, it facilitates observation of details that are averaged out in static FC and thus offers richer information for studying brain activities. On the other hand, it enables capture of spontaneously reoccurring functional connectivity patterns (i.e., FC states), which is essential for understanding the temporal variability in the intrinsic organization of the brain. Based on these advantages, researchers found that dynamic FC was a potential sensitive biomarker for neuropsychiatric disorders, such as Schizophrenia [19], Autism [20], and Parkinson's disease [21, 22]. The graph theory attributes are extended based on dynamic FC as well [23, 24]. However, to our knowledge, no study has examined the dynamic FC in MSA patients until now. To fill in the gap, this paper intends to combine the static and dynamic FC to focus on the following two questions: 1) whether and how MSA patients differ from healthy controls (HCs) in the static and dynamic FC at the whole-brain level; 2) whether such differences in static and dynamic FC can serve as potential biomarkers of MSA. Based on the previous studies and the pathology of MSA, we can hypothesize that alpha synuclein-positive GCI may disrupt the connectivity of DMN regions, sensorimotor cortex and cerebellum, destroy the network topology pattern in these regions, which are associated with clinical dysfunction. By using static and dynamic FC analysis, we can deeply understand the differences between MSA and controls, and possibly capture the most sensitive biomarker to differentiate the two groups.

In the current study, we propose a novel joint analysis method, investigating the network topology of static and dynamic FC based on the resting-state fMRI data of 24 MSA patients and 20 HCs. Firstly, we calculated four graph theory attributes based on static and dynamic FC. Secondly, statistical comparisons were performed and the correlations between features and clinical performance were measured in static and dynamic cases independently. Then, we identified features that are significantly abnormal and correlated with clinical scores in both static and dynamic cases, as well as static specific and dynamic specific features. Finally, we used the receiver operating characteristic (ROC) analysis to investigate whether the significantly different and correlated features can serve as predictors to distinguish MSA from HC.

## RESULTS

### Demographic and neuropsychological tests

Demographic and clinical characteristics are described in Table 1. No significant differences of gender, age, education, MMSE and MoCA scores were found between the MSA-c type and control groups. However, the MSA-c type group exhibited increased total UMSARS scores which refer to the severity of the disease.

**Table 1:** Clinical and demographical data.

	**MSA-C(n=24)**	**Control(n=20)**	***p*-Value**
Age, years	57.29±1.20	57.20±1.11	0.715
Gender, male/female	14/10	7/13	0.383
Education, years	13.80±0.48	13.75±0.49	0.192
Disease duration years	4.27±0.18	NA	
MMSE	27.20±0.45	27.30±0.42	0.374
MoCA	27.40±0.29	28.10±0.28	0.378
UMSARS-I	16.54±1.17	NA	
UMSARS-II	16.17±1.28	NA	
UMSARS- total	32.71±2.27	NA	
Over disability grade	2.49±0.25	NA	

### Graph attributes analysis

Statistical analysis on graph attributes identified a range of significantly different and correlated regions. A region is considered as significantly different if the non-parametric Mann Whitney test produced a p-value < 0.05. The correlation analysis between four graph theory attributes and total UMSARS scores selects regions that showed a p-value less than 0.05 in Kendall correlations. We identified regions and features that were significant (difference and correlation) in static-alone, dynamic-alone and both scenarios. Results are summarized in Table 2.

**Table 2:** Significant static and dynamic features.

**Graph theory attributes**	**Brain region**	**Brodmann area / Automated Anatomical Labeling atlas area**	**Static**				**Dynamic**			
			**Mean HC**	**Mean CMSA**	**Strength P**	**Kendall Correlation**	**Mean HC**	**Mean CMSA**	**Strength P**	**Kendall Correlation**
BC	Left dorsolateral prefrontal cortex	BA46	29.30	61.75	0.013	**0.336**	-	-	-	-
	Left Cerebellum_Crus9	AAL105	34.40	58.50	0.043	**0.370**	-	-	-	-
WD	Vermis_6	AAL112	24.60	21.99	0.027	**-0.292**	-	-	-	-
BC	Left frontal eye field	BA8	-	-	-	-	94.99	67.57	0.005	**0.314**
CCFS	Right ventral anterior cingulate cortex	BA24	-	-	-	-	0.4969	0.3735	0.002	**-0.396**
LE	Right Cerebellum_Crus1	AAL92	-	-	-	-	0.2420	0.2187	0.010	**-0.292**
LE	Right Cerebellum_Crus2	AAL94	0.2348	0.1985	0.001	**-0.314**	0.2498	0.2187	0.003	**-0.314**
	Right Cerebellum_Crus 6	AAL100	0.2356	0.2010	0.006	**-0.366**	0.2464	0.2213	0.009	**-0.351**
	Vermis_6	AAL112	0.2148	0.1961	0.029	**-0.366**	0.2309	0.2135	0.032	**-0.336**
WD	Right Cerebellum_Crus 6	AAL100	27.33	22.94	0.009	**-0.322**	28.51	25.21	0.014	**-0.329**

The p-value is obtained by non-parametric Mann-Whitney tests. BC: betweenness centrality; CCFS: clustering coefficient; LE: local efficiency; WD: weighted degree. Significant correlations were shown in bold (p < 0.05, uncorrected).

### Static and dynamic graph attributes

Among features identified from static graph attributes, BC at left dorsolateral prefrontal cortex (DLPFC, BA46) and left Cerebellum_Crus9 (AAL105), LE at right Cerebellum_Crus2 (AAL94), right Cerebellum_Crus6 (AAL100) and Vermis_6 (AAL112), WD at right Cerebellum_Crus6 (AAL100) and Vermis_6 (AAL112) showed significant difference and had significant correlation with clinical scores. Patients showed increased BC values and decreased LE and WD values at these regions Cerebellum_Crus. The correlations of BC with scores were all positive, whereas CCFS, LE and WD were negatively correlated with scores (Table 2).

For dynamic feature results, BC at left frontal eye field (BA8), CCFS at right VACC, LE at right Cerebellum_Crus1 (AR92), right Cerebellum_Crus2, right Cerebellum_Crus6 and Vermis_6, WD at right Cerebellum_Crus6 were both significantly different and correlated with scores. Patients showed reduced CCFS, LE and WD values at these regions, while with decreased BC at left frontal eye field. The correlations of BC with scores were positive, whereas CCFS, LE and WD were negatively correlated with scores (Table 2).

The stability of dynamic graph attributes was also examined. The stability of patients was higher than healthy controls in several regions for LE and WD, while healthy controls were more stable for CCFS. The betweenness centrality (BC), however, exhibited mixed result. Only BC at left frontal eye field was both significantly different in strength (p<0.005) and stability (p<0.004), as well as correlated with scores (Kendall taur = 0.333). BC of AD patients at left frontal eye field was more stable than healthy controls. The detailed statistics can be found in Supplementary Table 1

### Static and dynamic overlapping graph attributes

The intersection of static and dynamic significant features were LE at right Cerebellum_Crus2, right Cerebellum_Crus6 and Vermis_6, and WD at right Cerebellum_Crus6. As reported before, statistical tests results were consistent in static and dynamic FC. Patients showed reduced LE and WD values at these regions. The correlations of CCFS, LE and WD with scores were negative.

**Figure 1:**
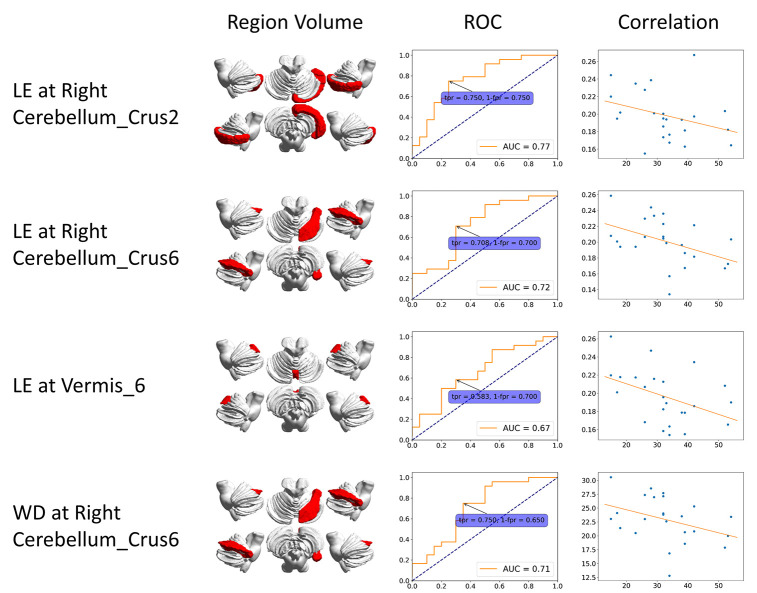
**ROC curve and correlation of static features.** The correlation is between clinical score (x-axis) and features (y-axis). BC: betweenness centrality; CCFS: clustering coefficient; LE: local efficiency; WD: weighted degree.

**Figure 2:**
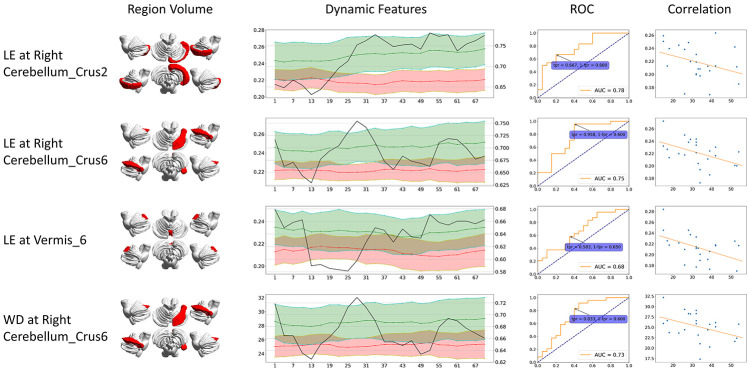
**Dynamic graph attributes curves with ROC AUC plots, ROC curve at the maximum ROC AUC time point, and correlation of features and clinical scores.** For Dynamic Features line plots, black line stands for ROC AUC (value on the right axis). Green stands for healthy controls and red stands for CMSA patients (value on the left axis). The ROC curve at the time point when AUC reached its maximum is shown. The correlation is between clinical score (x-axis) and features (y-axis). BC: betweenness centrality; CCFS: clustering coefficient; LE: local efficiency; WD: weighted degree.

In order to visualize dynamic graph attributes, we calculated the average and 95% confidence interval within each group at each time slice. Patients and healthy controls were shown as red and green respectively. The average value lies in the middle, bounded by the upper and lower 95% confidence interval curves. The correlation results of static and dynamic features are also plotted in Figures 1 and 2 respectively.

We plotted the receiver operating characteristic (ROC) curve based on each of the significant regions. Figure 1 shows results from static FC. The ROC curve of dynamic features was generated at each time slice and the area under curve (AUC) value was calculated. A black line represents the variation of AUC and is shown in the dynamic plots. The ROC curve related to the maximum AUC of dynamic features is shown in Figure 2.

Although the feature locations are the same, dynamic significant features generally had higher AUC compared to their static counterparts. The dynamic significant feature of LE at right Cerebellum_Crus2 reached the highest ROC AUC of 0.78, followed by dynamic significant feature of LE at right Cerebellum_Crus6 with ROC AUC of 0.75.

### Static and dynamic specific graph attributes

**Figure 3:**
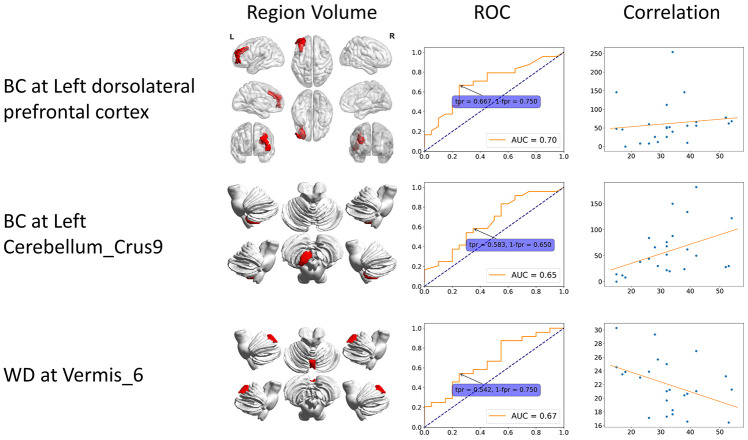
**Static specific significant features.** These features can only be identified from static functional connectivity. The 3D region volume was shown, as well as the ROC curve and correlation with clinical scores. The correlation is between clinical score (x-axis) and features (y-axis). BC: betweenness centrality; CCFS: clustering coefficient; LE: local efficiency; WD: weighted degree.

**Figure 4:**
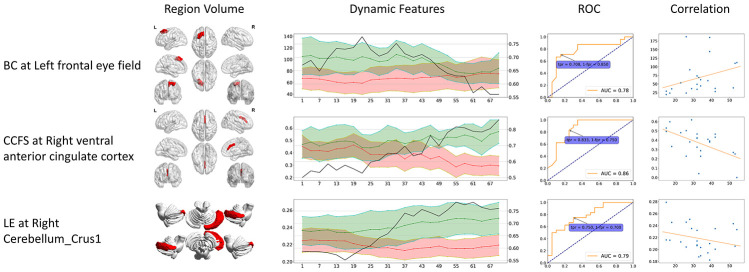
**Dynamic specific significant features.** These significant features can only be identified from dynamic functional connectivity. The 3D region volume was shown, as well as the dynamic features line plots. Green stands for healthy controls, red stands for CMSA patients (value on the left axis), and black line stands for ROC AUC (value on the right axis). The ROC curve at the time point when AUC reached its maximum is shown. The correlation is between clinical score (x-axis) and features (y-axis). BC: betweenness centrality; CCFS: clustering coefficient; LE: local efficiency; WD: weighted degree.

In order to investigate differences of static and dynamic graph attributes, we identified features that are significant only in static or dynamic cases. The static specific significant features include BC at left DLPFC, left Cerebellum_Crus9 and WD at Vermis_6, while dynamic specific significant features include BC at left frontal eye field, CCFS at right VACC, and LE at Cerebellum_Crus1. Patients had higher static BC at left DLPFC and left Cerebellum_Crus9, and lower static WD at Vermis_6. Static BC at left DLPFC and left Cerebellum_Crus9 were positively correlated with scores while static WD at Vermis_6 showed negative correlation. All dynamic specific significant features were lower in the patient group and had negative correlation except BC at left frontal eye field. The region volume, dynamic feature line plot, ROC curve and correlation of static and dynamic specific significant features are shown in Figures 3 and 4 respectively.

The dynamic specific significant features also showed higher AUC values compared to static specific significant features. Dynamic CCFS at right VACC reached the highest AUC of 0.86, higher than LE at right Cerebellum_Crus2 (AUC = 0.78).

### Logistic regression analysis

**Figure 5:**
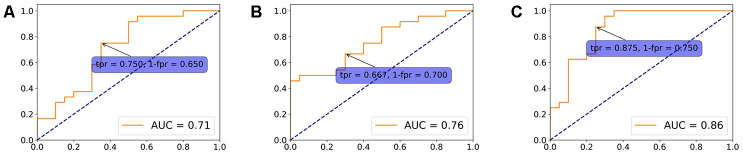
**Logistic regression model.** A logistic regression model using overlapping (**A**), static specific (**B**) and dynamic specific features (**C**) with regularization parameter C = 1. ROC curves were plotted to evaluate the classifier performance.

In order to assess the value of identified significant features in disease classification, we fitted a logistic regression model using static specific, dynamic specific and overlapping features with regularization parameter C = 1. ROC curves were plotted to evaluate the classifier performance (Figure 5). The model built with dynamic specific features reached the highest AUC of 0.86, with 87.5% sensitivity and 75.0% specificity.

## DISCUSSION

### Major findings

As the first attempt to investigate the FC dynamics of MSA at the whole-brain level, the present work analyzed the regional temporal variability of graph theory attributes related to MSA patients in comparison with HCs. Our main findings are as follows. First, among the features identified from both static and dynamic graph attributes, LE at right Cerebellum_Crus2, right Cerebellum_Crus6 and Vermis_6, and WD at right Cerebellum_Crus6 had significant correlations with total UMSARS scores. Second, when investigating the differences between static and dynamic graph attributes, we identified features that are significantly abnormal only in static or dynamic cases. The static specific significant features include BC at left DLPFC, left Cerebellum_Crus9 and WD at Vermis_6, while dynamic specific significant features include BC at left frontal eye field, CCFS at right VACC, and LE at right Cerebellum_Crus1. All the features had significant correlation with total UMSARS scores. Third, the ROC analysis revealed that among all static and dynamic features, dynamic specific CCFS at right VACC showed the largest area under the curve, implying that the dynamic functional change of VACC may be the key imaging marker for MSA diagnosis.

### Features identified from both static and dynamic graph attributes

Among the features identified from both static and dynamic graph attributes, some features were found to have significant correlations with total UMSARS scores in several cerebellar subregions. BC, CCFS, LE and WD are measures of regional functional “importance” based on the strength of a region’s connections with other regions and importance of the connected brain regions themselves. In our study, we found LE and WD in MSA patients were lower than healthy controls in the identified areas and showed mostly negative correlation. The decreased LE at right Cerebellum_Crus2, right Cerebellum_Crus6 and Vermis_6, and WD at right Cerebellum_Crus6 suggested that the accumulation of alpha-synuclein GCIs of the MSA may destroy specific networks including the DMN and cerebellum networks [8, 12].

Apart from the overlapping significant graph attributes in both static and dynamic, there are several static and dynamic specific graph attributes. The static specific significant features include increased BC at left DLPFC and left Cerebellum_Crus9, decreased WD at vermis_6, while dynamic specific significant features include decreased BC at left frontal eye field, decreased CCFS at right VACC, and decreased LE at right Cerebellum_Crus1. This discrepancy between static and dynamic graph attributes indicates that dynamic functional connectivity can reveal extra significant features. As ROC analysis reported below, some features showed a strong predictive value only in dynamic cases.

We also identified BC at left frontal eye field as the only feature that showed difference on dynamic stability and strength, as well as correlated with scores. Stability measures the amount of changes of a feature during a period of time [25]. BC at left frontal eye field in patients was more stable than healthy controls, meaning that the brain network's topology related to left frontal eye field of patients did not change as much as healthy controls. This network stiffness may be caused by MSA. The occurrence of BC at left frontal eye field in the set of significant difference and significant correlation dynamic features also implies the link of dynamic functional alteration, dynamic stability and clinical traits of MSA patients at left frontal eye field.

As the essential component of DMN, VACC was found to have lower CCFS in MSA patients than healthy controls. The DMN was a functional-anatomic network which is thought to be responsible for self-reflection, memory and stream-of-consciousness processing [26, 27]. The disruption of DMN was consistently demonstrated in Alzheimer’s disease (AD) by many resting-state fMRI studies, which contributed to the memory deficit of AD [28, 29]. However, with the gradually deeper understanding of DMN, researchers found that changes of DMN occurred in several other neurological disorders, including depression [30], autism spectrum disorders [25], schizophrenia [31], as well as in MSA [32]. These findings indicated that regions of DMN might be responsible for multiple functions beyond memory, including action, cognition, emotion, perception, interception and mental imaginary. Therefore, the disruption of DMN in the MSA-c type may result in the impairment of these functions. This result was consistent with the previous MSA studies, which also reported the disruption of DMN in the MSA-c type patients [8, 10, 12, 33].

The decreased LE at right Cerebellum_Crus1, right Cerebellum_Crus2, right Cerebellum_Crus6 and Vermis_6, WD at Vermis_6 and right Cerebellum_Crus6 were found in our study. To our knowledge, as the afferent fibers of cerebellum, cerebello-cortical circuit mainly comes from the opposite cerebellopontine nucleus and the inferior olivary nucleus, passing through the middle and lower cerebellar peduncles to the new cerebellum. And then, the cerebellar cortex sends the efferent fibers to dentate nuclei and forms the main body of the superior cerebellar peduncles, which project into the contralateral thalamus and cerebral cortex. From the view of the process of the cerebello-cortical circuit, many cerebellum regions play an important role in the network, which is responsible for the planning, balance, and coordination of motor functions. In this study, the disconnection within several cerebellum regions was consistent with the pathology of MSA-c type, which emphasizes on the cerebellum atrophy and dysfunction [34, 35]. In addition, these results matched well with several previous resting-state fMRI studies [8, 10, 12, 33].

In the present study, we found that BC in MSA patients was higher than healthy controls at left DLPFC and left Cerebellum_Crus9. We speculate that this strange result may be related to its compensatory effect. In addition, we found BC in MSA patients was lower than healthy controls in left frontal eye field, which is a part of the visual network. According to the established international diagnostic criteria of probable MSA defined by the American Academy of Neurology and American Autonomic Society [1], patients with MSA often show damage of visuospatial function. In addition, Kawai’s study [36] reported that patients with MSA showed severe involvement of visuospatial and constructional function compared with control subjects.

### The dynamic graph attributes analysis of ventral anterior cingulate cortex as biomarker

In the previous studies, most researchers used structural changes as biomarker to differentiate the MSA-c type and controls. For example, a previous study used the “hot cross bun” sign as biomarker to diagnose MSA-c type, yielding a high specificity of 97%, but its sensitivity was only 50% [37]. In the current study, to get a valuable imaging marker, we performed ROC analysis on all features identified from both static and dynamic graph attributes, finding that most of the dynamic significant features show higher AUC values than static significant features. When using the feature of CCFS at right VACC in dynamic graph attributes as the biomarker, we could differentiate the two groups at the cutoff value of 0.503, yielding a sensitivity of 83.3%, specificity of 75.0%, and reach the highest AUC of 0.86. This is a meaningful result which could be used as a valuable imaging marker for the early diagnosis of MSA-c type.

### The correlation between graph attributes and clinical performances

In this study, we found a close relationship between motor impairment (total UMSARS scores) and the static and dynamic graph attributes in several regions of the MSA patients, which suggested a clinical relevance of static and dynamic functional disruption in MSA. The altered static and dynamic FC in these regions might be used as imaging markers for tracking disease progression.

### Dynamic versus static graph attributes

In our work, we analyzed dynamic graph attributes based on dynamic functional connectivity (DFC) and incorporated static graph theory attributes to identify significant regions. To our knowledge, no study has examined DFC in MSA patients before. Compared with static functional connectivity, DFC measures functional variation within a time slice of the scanning session and can reveal transient characteristics of brain activities that might be averaged out in static FC. We adopted a sliding-window approach with window length = 100 TR and step size = 3 TR. Our previous research proved that this combination can extract as much features while minimizing computation costs [38]. It is, however, an open question regarding the selection of sliding-window parameters and other methods to estimate dynamic functional connectivity [39–41].

The analysis results showed that dynamic graph attributes shared similar significant features with static graph attributes, as well as identifying dynamic specific significant features. The overlapping significant features were similar between static and dynamic cases, implying that dynamic graph attributes can preserve stable features identified by static analysis. On the other hand, dynamic analysis revealed significant features that were especially useful at classifying patients, reaching the top ROC AUC of 0.86. It can be seen from the dynamic attributes line plot of CCFS at right VACC that the feature distributions of two groups were mixed at first, and split away as time varies. This implies that such high AUC value can only be identified using dynamic functional connectivity. As a result, dynamic analysis can not only identify most significant features as static analysis does, but also reveal extra features that could possibly be used as biomarker for disease classification.

### Future considerations

There are still some issues to be addressed. First, recent studies have paid more attention to MSA-p type. In the future, exploring MSA different subtypes (parkinsonian and cerebellar variants) would provide valuable biomarkers for the early differential diagnosis of the disease. Second, in this study, we mainly focused on the motor function changes of MSA, as measured by UMSARS. Some MSA patients might present cognitive dysfunction with the disease progresses. In the future, we will collect more samples and classify MSA as two groups according to cognitive performances. We will analyze network attributes to compare the differences between MSA with and without cognitive decline. Third, the sample size is relatively small, and in the future, we will continue to expand the sample size. More samples could also validate statistical analysis methods such as stepwise multivariate logistic models. Finally, in the current study, when measuring dynamic functional connectivity (d-FC), we adopted a sliding-window method, with window length = 100 TR (200 s) and step size = 3 TR (6 s), as a previous research proved that this combination can extract as much features while minimizing computation costs [38]. And the analysis was performed at each brain region. In the future, we can adopt different sliding-window methods for comparison as well as performing global comparison of graph attributes, which might reveal other evidence on the implications of MSA.

## CONCLUSION

In conclusion, by using static and dynamic functional connectivity, we identified significant graph attributes in several specific regions in MSA patients. For the overlapped results, disrupted connectivity in several cerebellar subregions was found in MSA patients relative to controls. In addition, we also identified some static and dynamic specific significant graph attributes. Further ROC analysis revealed that dynamic CCFS at right VACC could be used as the most valuable imaging marker for the early diagnosis of MSA, indicating the advance of dynamic functional connectivity analysis. These findings provided new evidence for the disconnection syndrome of MSA and emphasized the importance of dynamic functional connectivity analysis in deepen the understanding of the disease.

## MATERIALS AND METHODS

### Participants

Twenty four MSA c-type patients and twenty controls were recruited at the clinic of Dongfang Hospital of Beijing University of Chinese Medicine. The two groups were matched for age and gender and all the participants are right-handed. The diagnosis of MSA was according to the established international diagnostic criteria of probable MSA defined by the American Academy of Neurology and American Autonomic Society [1]. All subjects were evaluated by complete physical and neuropsychological examinations including mini-mental state examination (MMSE), Montreal Cognitive Assessment (MoCA), and Unified Multiple System Atrophy Rating Scale (UMSARS). The clinical examinations were performed on the day before fMRI scanning.

The inclusion criteria for controls were as follows: (1) there were no neurological or psychiatric disorders including obsessive disorder, anxiety disorder, schizophrenia, depression, epilepsy and so on; (2) there were lack of significant cognitive decline (MMSE score > 24);(3) there were no neurological deficiencies including visual or hearing loss; (4) there were no treatment with deep brain stimulation or operation (5) there were no evidence of movement disorder, vascular brain lesions, brain tumor, and/or marked cortical and/or subcortical atrophy on MRI scan.

The exclusion criteria for the subjects were as follows: The subjects of hemorrhage, infarction, tumors, trauma, or severe white matter hyper-intensity were excluded from the study. Clinical and demographic information of the subjects was shown in Table 1.

All subjects gave written informed consent in accordance with the Declaration of Helsinki. The protocol was approved by the Medical Research Ethical Committee of Dong fang Hospital of Beijing University of Chinese Medicine.

### MRI acquisition protocol

MRI data acquisition was performed on a GE 3.0T Discovery 750 scanner. Foam padding and headphones were used to control head motion and scanner noise. The resting-state fMRI data was acquired by the following parameters with 6 minutes: repetition time (TR)/echo time (TE)/flip angle (FA) = 2000 ms/30 ms/90°, field of view (FOV) = 24 × 24 cm^2^, resolution = 64 × 64 matrix, number of slices = 36, thickness = 3 mm, gap = 1 mm, voxel size = 3.75 × 3.75 × 3 mm^3^, and bandwidth = 2232 Hz/pixel. In total, 6480 images were acquired for resting state fMRI. All the participants were instructed to keep their eyes closed, move as little as possible, think of nothing in particular, and stay awake during the scans.

### Image preprocessing

The preprocessing of fMRI data was carried out using DPARSFA (V4.3) [42] and SPM12 (V6906). We discarded the first 10 time points to let subjects be familiar with the scanning environment. Slice timing correction was then performed. Head motion was corrected before normalizing the image to a 2mm-isotropic BOLD EPI template in the Montreal Neurological Institute (MNI) 152 standard space. The image was resampled to 3-mm isotropic voxels and spatially smoothed by a Gaussian kernel with 4mm full-width half-maximum (FWHM). Then, we removed the linear trend and nuisance covariates, including head motions, cerebral fluid, white matter and the global signal. Finally, the signal time course was filtered to keep signals within 0.01-0.08Hz.

**Figure 6:**
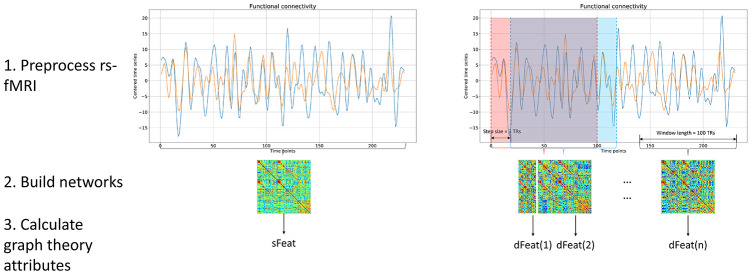
**Processing pipeline.** Both static and dynamic functional connectivity was calculated based on BOLD fMRI signal. For static functional connectivity, the pair-wise Pearson correlation between two regions was obtained using the whole time series. For dynamic functional connectivity, we utilized the sliding window method to produce a range of functional networks. After network construction, graph theory attributes were calculated based on each functional networks, yielding sFeat for static feature, and dFeats for dynamic features.

The data processing was carried out using an in-house software, Multi-Modal Data Processing System (MMDPS) [43]. We applied both static and dynamic functional connectivity (s-FC and d-FC) to evaluate functional changes in MSA patients. Graph theory attributes were also calculated based on static and dynamic FC. The whole processing pipeline is shown in Figure 6.

### Network construction

During network construction, we adopted a brain atlas to define nodes and constructed brain network for each participant. The atlas consists of 84 whole-brain Brodmann areas [44] and the cerebellum parcellation from the Automated Anatomical Labeling atlas with 26 regions [45]. Since cerebellum is actively involved in the progression of CMSA, we hope the inclusion of it could reveal disease-specific features. For static functional connectivity (s-FC), the BOLD time course was averaged within each brain area and Pearson correlation coefficients were calculated for each pair of regions, representing the functional connectivity between these two nodes in the network.

When measuring dynamic functional connectivity (d-FC), we adopted a sliding-window method, with window length = 100 TR (200 s) and step size = 3 TR (6 s). A previous research proved that this combination can extract as much features while minimizing computation costs [38]. The preprocessed data contained 170 TRs so the sliding window method yielded 24 dynamic networks for each subject.

### Graph theory attributes calculation

We calculated four graph theory attributes to reflect the topology of brain networks. The graph theory attributes are all associated with nodes in networks. The betweenness centrality (BC) of a node is the fraction of all shortest paths in the network that pass through the given node, indicating the nodal ability of information flow throughout the network. The clustering coefficient (CCFS) is the fraction of triangles around a node, measuring the tendency of all nodes in its neighborhood to form a cluster. The local efficiency (LE) of a node is measured as the average sum of inverse shortest path of other nodes to the given node. It quantifies the efficiency of a network to transfer information. And the weighted degree (WD) of a node is the sum of its connectivity strength. Among these four graph theory attributes, CCFS and LE fall in range [0, 1]. BC can be normalized to [0, 1] by dividing by (*n* − 1) × (*n* − 2), where n is the

These graph theory attributes were calculated based on individual node in each brain network, including both static (s-BC, s-CCFS, s-LE and s-WD) and dynamic networks (d-BC, d-CCFS, d-LE and d-WD). The static network produced one set of graph attributes for each subjects, while dynamic networks yielded graph attributes at each time slice. As a result, 24 data points were calculated for each subject and each attribute. These data points were arranged by time order, forming a fluctuating series of attributes for a participant during the scanning interval. We also measured the stability of dynamic graph attributes using methods proposed in [46]. All graph theory attributes were normalized to range [0, 1] before calculating stability.

$$dFeat_{Stability}=1-\frac{1}{T-1}\overset{T-1}{\underset{t=1}{\sum }}|Feat\left(t+1\right)-Feat\left(t\right)|$$

### Statistical analysis and feature selection

**Figure 7:**
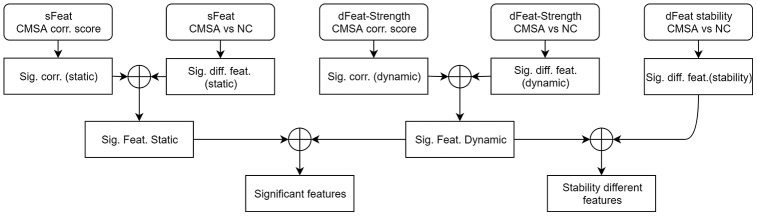
**Statistical analysis procedure.** At the first stage, comparison and correlation analysis was performed for static and dynamic features separately. At the second stage, significantly different and correlated features were combined. At the third stage, significant features found by static and dynamic functional connectivity were merged, as well as incorporating dynamic stability measurements. The circle-plus sign stands for intersection. sFeat, static feature; dFeat, dynamic feature; corr., correlate; Sig., significant; Feat, feature; diff, difference.

The statistical analysis procedure, including comparison and correlation, is summarized in Figure 7. At the first stage, static and dynamic features were analyzed separately and the same procedure was repeated for each one of the four graph attributes. For dynamic graph attributes, since each subject was associated with multiple data points at a range of time slices, we firstly calculated the strength of the dynamic attributes using methods described in [46] before performing comparison and correlation.

$$dFeat_{Strength}=\frac{1}{T}\overset{T}{\underset{t=1}{\sum }}Feat\left(t\right)$$

Since the features were not normally distributed, and the sample size was relatively small, we performed non-parametric Mann-Whitney tests between MSA patients and NCs. Significant regions (p < 0.05, uncorrected) were selected, forming a set of significantly different features (Sig. diff. feat. static / dynamic). The comparison of dynamic stability between patients and healthy controls was also performed. On the other hand, the Kendall correlation coefficients were calculated between features and total UMSARS scores. Areas that had a significant correlation (p < 0.05, uncorrected) were extracted, forming a set of significantly correlated features (Sig. corr. static / dynamic).

Then, at the second stage, the significantly different and correlated sets of static and dynamic graph attributes were intersected, yielding static and dynamic significant features (Sig. Feat. static / dynamic). At the third stage, the static and dynamic significant features were further intersected, identifying features that were significant in both static and dynamic cases. Static and dynamic specific features were left out at this stage. The stability of dynamic graph attributes were also incorporated into the dynamic significant features and the intersection gave stability different features.

## Supplementary Material

Supplementary Table 1
