# Supplementary material for: Abnormal static and dynamic functional connectivity of resting-state fMRI in multiple system atrophy
Source: Aging (Albany NY). 2020 Aug 27;12(16):16341–56. doi: 10.18632/aging.103676 (PMC7485713; doi:10.18632/aging.103676)
Supplement: Supplementary Table 1 [file aging-12-103676-s001..docx]

Supplementary Table 1. Dynamic stability comparison results.

| **BC** |  |  |  |  |  |  |  |
| --- | --- | --- | --- | --- | --- | --- | --- |
| tick | stat | statp | CMSA_mean | CMSA_std | NC_mean | NC_std | CMSA - NC |
| L7 | 152 | 0.019568 | 0.999012 | 0.000286 | 0.999223 | 0.000326 | -0.00021 |
| L8 | 125.5 | 0.003598 | 0.999164 | 0.000304 | 0.998887 | 0.000292 | 0.000277 |
| L20 | 153.5 | 0.0213 | 0.999034 | 0.000332 | 0.999203 | 0.000433 | -0.00017 |
| L22 | 168.5 | 0.047098 | 0.998918 | 0.00048 | 0.99916 | 0.00034 | -0.00024 |
| L24 | 141 | 0.010114 | 0.999133 | 0.000415 | 0.999415 | 0.000202 | -0.00028 |
| L34 | 160 | 0.030449 | 0.999412 | 0.000242 | 0.999195 | 0.000417 | 0.000217 |
| L35 | 166.5 | 0.042648 | 0.999176 | 0.000462 | 0.999404 | 0.000354 | -0.00023 |
| L39 | 105 | 0.000761 | 0.998874 | 0.000315 | 0.999236 | 0.00029 | -0.00036 |
| L46 | 162.5 | 0.034744 | 0.999159 | 0.000476 | 0.999386 | 0.000212 | -0.00023 |
| L47 | 157 | 0.025894 | 0.999202 | 0.000344 | 0.99897 | 0.000389 | 0.000232 |
| R1 | 149 | 0.016436 | 0.998928 | 0.000359 | 0.999175 | 0.000334 | -0.00025 |
| R2 | 150.5 | 0.017941 | 0.998833 | 0.000377 | 0.999099 | 0.000272 | -0.00027 |
| R8 | 168.5 | 0.047069 | 0.999259 | 0.000228 | 0.998987 | 0.000485 | 0.000272 |
| R22 | 160 | 0.030459 | 0.998996 | 0.000349 | 0.999174 | 0.000324 | -0.00018 |
| R28 | 162.5 | 0.034735 | 0.999159 | 0.000364 | 0.99933 | 0.000284 | -0.00017 |
| AL97 | 152.5 | 0.020131 | 0.999127 | 0.000321 | 0.99877 | 0.000628 | 0.000357 |
| AR100 | 149.5 | 0.016929 | 0.999093 | 0.000263 | 0.998825 | 0.00043 | 0.000268 |
| AL101 | 167 | 0.043707 | 0.999361 | 0.000291 | 0.999173 | 0.000376 | 0.000188 |
| **CCFS** |  |  |  |  |  |  |  |
| tick | stat | statp | CMSA_mean | CMSA_std | NC_mean | NC_std | CMSA - NC |
| L5 | 169.5 | 0.049474 | 0.933731 | 0.036851 | 0.91415 | 0.04115 | 0.01958 |
| L8 | 160 | 0.030477 | 0.940551 | 0.032713 | 0.956228 | 0.020326 | -0.01568 |
| L27 | 162 | 0.033868 | 0.907964 | 0.05076 | 0.924732 | 0.072996 | -0.01677 |
| L38 | 158 | 0.027368 | 0.92782 | 0.054061 | 0.9565 | 0.022512 | -0.02868 |
| L45 | 141 | 0.010125 | 0.92996 | 0.031331 | 0.950429 | 0.02259 | -0.02047 |
| R8 | 151 | 0.018491 | 0.930265 | 0.027118 | 0.944076 | 0.042668 | -0.01381 |
| R32 | 154 | 0.021939 | 0.925536 | 0.048381 | 0.94932 | 0.041469 | -0.02378 |
| R37 | 166.5 | 0.042632 | 0.918038 | 0.04419 | 0.933012 | 0.053897 | -0.01497 |
| R42 | 160 | 0.030477 | 0.938753 | 0.03788 | 0.957583 | 0.019308 | -0.01883 |
| R44 | 142 | 0.010778 | 0.909452 | 0.052215 | 0.942488 | 0.036366 | -0.03304 |
| AR92 | 143 | 0.011467 | 0.926233 | 0.041114 | 0.949797 | 0.035577 | -0.02356 |
| AL93 | 155.5 | 0.023854 | 0.923048 | 0.050481 | 0.945473 | 0.041043 | -0.02243 |
| AR94 | 112 | 0.001327 | 0.926634 | 0.048035 | 0.954414 | 0.019821 | -0.02778 |
| AL95 | 181 | 0.083969 | 0.938255 | 0.028948 | 0.907939 | 0.059079 | 0.030316 |
| AR100 | 158 | 0.027368 | 0.926052 | 0.041987 | 0.948492 | 0.023224 | -0.02244 |
| AR102 | 167 | 0.04374 | 0.919992 | 0.03311 | 0.930326 | 0.053186 | -0.01033 |
| AR108 | 168.5 | 0.047058 | 0.906059 | 0.049357 | 0.929527 | 0.05319 | -0.02347 |
| AR112 | 165.5 | 0.040557 | 0.91252 | 0.054934 | 0.934891 | 0.046076 | -0.02237 |
| AR116 | 153.5 | 0.021289 | 0.900742 | 0.07646 | 0.93814 | 0.054183 | -0.0374 |
| **LE** |  |  |  |  |  |  |  |
| tick | stat | statp | CMSA_mean | CMSA_std | NC_mean | NC_std | CMSA - NC |
| L10 | 138 | 0.00837 | 0.995468 | 0.00128 | 0.994816 | 0.0011 | 0.000652 |
| L24 | 162 | 0.033873 | 0.9956 | 0.001031 | 0.994899 | 0.001304 | 0.000701 |
| L25 | 160 | 0.030477 | 0.995373 | 0.001269 | 0.994746 | 0.001375 | 0.000628 |
| L26 | 160 | 0.030477 | 0.995372 | 0.001167 | 0.994608 | 0.00125 | 0.000764 |
| L28 | 162 | 0.033873 | 0.995755 | 0.000882 | 0.995205 | 0.001017 | 0.00055 |
| L34 | 120 | 0.002426 | 0.99559 | 0.000863 | 0.99467 | 0.001018 | 0.00092 |
| L45 | 138 | 0.00837 | 0.99581 | 0.00083 | 0.995108 | 0.00105 | 0.000702 |
| L48 | 167 | 0.04374 | 0.995796 | 0.000983 | 0.995367 | 0.000877 | 0.00043 |
| R3 | 162 | 0.033873 | 0.995443 | 0.001236 | 0.994889 | 0.001158 | 0.000554 |
| R4 | 169 | 0.048286 | 0.995626 | 0.001159 | 0.994995 | 0.001108 | 0.000631 |
| R6 | 154 | 0.021939 | 0.995585 | 0.001254 | 0.994713 | 0.001513 | 0.000872 |
| R10 | 167 | 0.04374 | 0.995743 | 0.000825 | 0.99507 | 0.001429 | 0.000673 |
| R21 | 160 | 0.030472 | 0.995724 | 0.001081 | 0.994994 | 0.001217 | 0.000729 |
| R24 | 134 | 0.006447 | 0.995682 | 0.000731 | 0.994394 | 0.00175 | 0.001287 |
| R26 | 146 | 0.013769 | 0.995661 | 0.000614 | 0.995114 | 0.001039 | 0.000547 |
| R27 | 156 | 0.024527 | 0.995684 | 0.00136 | 0.994947 | 0.001325 | 0.000737 |
| R34 | 151 | 0.018491 | 0.995411 | 0.001126 | 0.994473 | 0.001411 | 0.000937 |
| R39 | 154 | 0.021939 | 0.995725 | 0.000735 | 0.995268 | 0.000853 | 0.000458 |
| R46 | 161 | 0.032138 | 0.995762 | 0.000967 | 0.995063 | 0.00117 | 0.000699 |
| AR98 | 149.5 | 0.016944 | 0.995582 | 0.000934 | 0.995083 | 0.000772 | 0.000499 |
| AL99 | 157 | 0.025915 | 0.995746 | 0.001092 | 0.995051 | 0.001187 | 0.000694 |
| AL113 | 153 | 0.020734 | 0.995502 | 0.001134 | 0.994596 | 0.001379 | 0.000906 |
| AR114 | 149 | 0.016458 | 0.995169 | 0.001116 | 0.99435 | 0.001334 | 0.000819 |
| AL115 | 139 | 0.008923 | 0.995483 | 0.001217 | 0.99456 | 0.001316 | 0.000922 |
| **WD** |  |  |  |  |  |  |  |
| tick | stat | statp | CMSA_mean | CMSA_std | NC_mean | NC_std | CMSA - NC |
| L10 | 133 | 0.006033 | 0.994796 | 0.001711 | 0.994034 | 0.001141 | 0.000762 |
| L24 | 159 | 0.028887 | 0.995026 | 0.001156 | 0.994157 | 0.001637 | 0.000869 |
| L25 | 169 | 0.048286 | 0.994838 | 0.001514 | 0.993979 | 0.001826 | 0.000859 |
| L28 | 153 | 0.020734 | 0.995311 | 0.001121 | 0.994625 | 0.001221 | 0.000686 |
| L32 | 154 | 0.021939 | 0.994922 | 0.001581 | 0.994212 | 0.001258 | 0.000711 |
| L34 | 137 | 0.007847 | 0.995042 | 0.001173 | 0.993745 | 0.001647 | 0.001297 |
| L36 | 167 | 0.04374 | 0.995178 | 0.001428 | 0.994558 | 0.001136 | 0.00062 |
| L45 | 146 | 0.013769 | 0.995286 | 0.001201 | 0.994261 | 0.001436 | 0.001025 |
| R6 | 166 | 0.0416 | 0.994783 | 0.001501 | 0.993961 | 0.001776 | 0.000822 |
| R10 | 153 | 0.020734 | 0.995171 | 0.000951 | 0.994084 | 0.001845 | 0.001086 |
| R21 | 159 | 0.028887 | 0.99511 | 0.001411 | 0.994291 | 0.001317 | 0.000819 |
| R24 | 131 | 0.005273 | 0.995246 | 0.000781 | 0.993698 | 0.002029 | 0.001548 |
| R26 | 120 | 0.002426 | 0.995115 | 0.000972 | 0.994149 | 0.001139 | 0.000966 |
| R27 | 157 | 0.025915 | 0.995042 | 0.001609 | 0.994387 | 0.001575 | 0.000655 |
| R34 | 152 | 0.019585 | 0.99488 | 0.001502 | 0.993781 | 0.001781 | 0.001099 |
| R39 | 110 | 0.001135 | 0.995198 | 0.00091 | 0.99446 | 0.000844 | 0.000738 |
| R46 | 149 | 0.016458 | 0.995245 | 0.001069 | 0.994384 | 0.001293 | 0.00086 |
| AR96 | 148 | 0.015515 | 0.995246 | 0.001263 | 0.994538 | 0.001125 | 0.000708 |
| AL97 | 168 | 0.045968 | 0.994846 | 0.001479 | 0.994343 | 0.001234 | 0.000502 |
| AR98 | 152 | 0.019585 | 0.994994 | 0.001171 | 0.994307 | 0.001174 | 0.000687 |
| AL99 | 162 | 0.033873 | 0.995177 | 0.00132 | 0.994311 | 0.001755 | 0.000866 |
| AR106 | 162 | 0.033873 | 0.9951 | 0.000962 | 0.994289 | 0.001295 | 0.000812 |
| AL113 | 128 | 0.004293 | 0.995229 | 0.001324 | 0.993699 | 0.001791 | 0.00153 |
| AR114 | 148 | 0.015515 | 0.994583 | 0.00149 | 0.993554 | 0.001729 | 0.001029 |
| AL115 | 140 | 0.009507 | 0.995052 | 0.001288 | 0.993809 | 0.001811 | 0.001243 |

Note: All features showing significant difference (Mann Whitney p < 0.05) are shown. Tick stands for Brodmann areas or the cerebellum parcellation in AAL. stat: Mann Whitney statistics; statp: p-value; CMSA_mean: average stability in CMSA group; CMSA_std: standard deviation of stability in CMSA group; NC_mean, NC_std: similar to CMSA; CMSA-NC: the difference of mean stability; BC: betweenness centrality; CCFS: clustering coefficients; LE: local efficiency; WD: weighted degree.
